# A Novel Polysaccharide From *Chuanminshen violaceum* and Its Protective Effect Against Myocardial Injury

**DOI:** 10.3389/fnut.2022.961182

**Published:** 2022-07-14

**Authors:** Peng He, Mi Zhang, Meng Zhao, Mengyao Zhang, Benxu Ma, Hongyu Lv, Yantao Han, Dingtao Wu, Zhangfeng Zhong, Wenwen Zhao

**Affiliations:** ^1^School of Basic Medical Sciences, Qingdao University, Qingdao, China; ^2^School of Nursing, Qingdao University, Qingdao, China; ^3^The Affiliated Qingdao Central Hospital of Qingdao University, The Second Affiliated Hospital of Medical College of Qingdao University, Qingdao, China; ^4^Key Laboratory of Coarse Cereal Processing (Ministry of Agriculture and Rural Affairs), Sichuan Engineering & Technology Research Center of Coarse Cereal Industralization, School of Food and Biological Engineering, Chengdu University, Chengdu, China; ^5^Macau Centre for Research and Development in Chinese Medicine, Institute of Chinese Medical Sciences, University of Macau, Zhuhai, Macao SAR, China

**Keywords:** *Chuanmingshen violacenm*, polysaccharide, antioxidant activity, myocardial ischemia-reperfusion, ferroptosis

## Abstract

We isolated and purified a novel polysaccharide from the root of *Chuanminshen violaceum*, namely, *Chuanminshen violaceumis* polysaccharide (CVP) and confirmed its structure and molecular weight. Furthermore, *in vivo* experiment, CVP’s protective effect against myocardial ischemia-reperfusion (I/R) injury in mice was evidenced by significantly reducing I/R-induced myocardial infarction (MI) size, decreasing the secretion of heart damage biomarkers, and improving cardiac function. Then, the myocardial anoxia/reoxygenation (A/R) injury model was established to mimic reperfusion injury. Noticeably, ferroptosis was the major death manner for A/R-damaged H9c2 cells. Meanwhile, CVP significantly inhibited ferroptosis by decreasing intracellular Fe^2+^ level, enhancing GPX4 expression, and suppressing lipid peroxidation to confront A/R injury. In conclusion, CVP, with a clear structure, ameliorated I/R injury by inhibiting ferroptosis.

## Highlights

- A polysaccharide namely CVP was isolated from the roots of *Chuanminshen violaceum*.
- CVP’ structure and molecular weight were confirmed.
- CVP showed a protective effect against myocardial I/R injury.
- Suppressing ferroptosis was the mechanism for CVP alleviating myocardial I/R damage.

## Introduction

Ischemic heart disease is a leading cause of death worldwide (1). Clinically, prolonged ischemia can lead to myocardial infarction (MI), and reperfusion is considered one preferred effective strategy to salvage ischemic myocardium (2) with primary means of percutaneous coronary intervention (PCI) (3) and coronary artery bypass grafting (CABG) (4, 5). Meanwhile, restoration of blood supply accompanied by a large amount of reactive oxygen species (ROS), severe calcium overload, and a series of inflammatory reactions could further aggravate myocardial tissue damage, which is called myocardial ischemia-reperfusion (I/R) injury (6, 7). Therefore, inhibition of oxidative stress and inflammatory response is widely accepted as the optimum therapeutic strategy for I/R injury (8). However accumulated clinical feedback reveals that serious side effects remain a vital challenge in dealing with oxidative damage, such as acute kidney injury (9), arrhythmia (10, 11), hypertension (12), sepsis (13), and bowel infarct (14). Therefore, there is an increasing demand for potential natural products with lower toxicity and fewer side effects. Herein phenolic compounds and polysaccharides are ideal candidates.

Polysaccharides, derived from a wide range of natural products, own mild properties and exhibit various bioactivities, such as immune-enhancing, anti-inflammatory, anti-oxidative, anti-cancer, and anti-aging effects (15–20). The multifunctional polysaccharides have been paid significant attention as medicines (21), cosmetics, and food (22). Recently, more and more documents reveal prominent protective effects of polysaccharides against cardiovascular disease, such as atherosclerosis (23), MI (24), and heart failure (25, 26). Polysaccharide PZMP2-1 from Ziziphus Jujuba cv. Muzao demonstrates excellent antioxidant activity, especially on scavenging 1,1-diphenyl-2-picrylhydrazyl (DPPH) and hydroxyl radicals, and has a potential for treating cardiovascular diseases (27). *Ganoderma lucidum* polysaccharide (GLPs) isolated from *Ganoderma lucidum* significantly reduces oxidative stress and apoptosis of myocardial cells and thus exhibits a protective effect on I/R injury (28). In addition, an increasing number of studies have indicated that the non-starch polysaccharides from *Chuanmingshen violaceumis* have commendable antioxidant activity and immune-mediated activity (29). However, there are limited reports on detailed mechanisms of polysaccharide intervention. In our study, a novel polysaccharide named *Chuanminshen violaceumis* polysaccharide (CVP) was isolated from the root of *Chuanminshen violaceumis*, one traditional Chinese medical herb, and then its effects on myocardial I/R injury and related mechanisms were elaborated both *in vivo* and *in vitro*.

Compared with ischemic injury, I/R injury causes paradoxical exacerbation of cellular dysfunction and death once restored blood flow to the ischemic area. Although I/R injury is a multi-factorial process resulting in extensive tissue destruction, precise pathological mechanisms are still unclear. Ferroptosis, a new form of regulated cell death was verified, plays a significantly detrimental role in a series of I/R models, such as intestinal I/R injury (30), renal I/R injury (31), and myocardial I/R injury (32, 33). In addition, several reports confirmed the contribution of pyroptosis to myocardial damage by targeting NLRP3/caspase-1 inflammatory signaling (34, 35). More research is needed on elucidating the cell death manners.

In all, CVP’s cardioprotective effect in I/R mice and related mechanisms based on H9c2 cells under A/R injury were explored in our study. The present study may provide valuable insight for preventing myocardial damage by targeting natural polysaccharides.

## Materials and Methods

### Materials and Chemicals

*Chuanminshen violaceumis*’ dry root was from Chengdu herbal market and then it was identified by a third-party verification at Sichuan Agricultural University. All standard monosaccharides (D-glucose, D-galactose, D-galacturonic acid, D-mannose, L-arabinose, and L-rhamnose), sulfuric acid, and phenol were obtained from Shanghai Aladdin Biochemical Polytron Technologies Inc. (Shanghai, China). All other mentioned reagents in the study were of analytical grade and got from Sigma-Aldrich (United States); both Dulbecco’s modified Eagle’s medium (DMEM) and fetal bovine serum (FBS) were bought from Life Technologies/Gibco Laboratories (Grand Island, NY, United States); Cytokine ELISA kits [interleukin-6 (IL-6) and tumor necrosis factor (TNF-α)] were purchased from NeoBioscience (Shenzhen, China); and IM54, ZVAF-FMK, ferrostatin-1 (Fer-1), and Hoechst 33258 were got from Selleck Chemicals (United States). Antibodies for GAPDH, GPX4, and SLC7A11 were purchased from Cell Signaling Technology (United States). C11 BODIPY 581/591 was purchased from Invitrogen (United States). ELISA Kit for cardiac troponin I (cTnI) was got from Cusabio Biotechnology Co., Ltd. (Wuhan, China). All other chemicals were purchased from Sigma Aldrich (United States).

### Extraction and Purification of *Chuanminshen violaceumis* Polysaccharide

Different polysaccharides were obtained according to our published in-house protocol (36). Briefly, dried *Chuanminshen violaceum* (20 g) was washed with 80% ethanol (40 ml) three times to remove small organic molecules and coloring matter. Then sediments were extracted twice with 400 ml of H_2_O adding 20 U/ml heat-stable α-amylase at 80°C with continuous stirring for 2 h. Obtained supernatants were further purified with highly efficient α-amylase (40,000 U/g) and concentrated with 70% (v/v) ethanol. After centrifugation, crude polysaccharides were got from precipitations and dialyzed at a cutoff of 3,000–30,000 Da to get the polysaccharide with a molecular weight of less than 3,000 Da, namely, CVP.

### Structure Characterization of *Chuanminshen violaceumis* Polysaccharide

#### Chemical Analysis of *Chuanminshen violaceumis* Polysaccharide

The homogeneity of CVP was determined with TSK-GEL G4000PWxl (TOSHO, Japan, 300 mm × 7.8 mm) and liquid chromatography (LC20-A, Shimadzu, Kyoto, Japan). Polyethylene glycol standards with different molecular weights (600, 2,000, 44,200, 146,000, 580,000, and 903,000 Da) were used to prepare the calibration curve. Fourier transform infrared (FT-IR) spectrum of CVP was recorded between 4,000 and 450 cm^–1^ with PerkinElmer Spectrum™ 100 FT-IR Spectrometer.

#### Monosaccharide Contents of *Chuanminshen violaceumis* Polysaccharide

Preliminary identification of monosaccharides isolated from CVP was performed by high-performance liquid chromatography (HPLC). Briefly, 4.0 mg polysaccharide was hydrolyzed with 2 ml of 2 M trifluoroacetic acid at 95°C for 10 h. Then hydrolysate was dissolved in methanol (1 ml) and then a rotary vacuum evaporator was used to evaporate the solvent. Subsequently, 1-phenyl-3-methyl-5-pyrazolone (PMP) was added to react with the hydrolysate. Then the mixture was kept at 70°C for 100 min and neutralized with a hydrochloric acid solution. After shaking with chloroform (1 ml) vigorously, the organic layer was discarded. Subsequently, the solution was filtered through a 0.22 μm membrane for HPLC analysis. At the same time, standard monosaccharides containing Ara, GalA, Gal, GlcA, Glc, Man, Rha, and Xyl were carried out. PMP derivatives were analyzed by an Agilent 1260 Series LC System (Agilent Technologies, Palo Alto, CA, United States) with a ZORBAX Eclipse XDB-C18 column (id 5 μm, 4.6 × 250 mm, Agilent Technologies Inc., CA, United States). The mobile phase consisted of a mixture of aqueous 0.1 M phosphate buffer (pH = 6.7) and acetonitrile (83:17, v/v).

#### Methylation Analysis

Methylation analysis of CVP was performed according to the previously reported method with a minor difference. Briefly, 10 mg of CVP was joined into dimethyl sulfoxide (DMSO) with dry NaOH powder. After the mixture was dissolved by strong ultrasonic, CH_3_I was added for reaction for 2 h under a nitrogen atmosphere. Then the sample was extracted with chloroform and the organic phase was separated to obtain the methyl sample. Subsequently, the reaction mixture was degassed with argon for 15 min and heated to reflux with stirring for 16 h. The final sample was hydrolyzed with trifluoroacetic acid (TFA), acetylated with AC_2_O, and analyzed by gas chromatography-mass spectrum (GC-MS; Agilent 6890A-5975C, Agilent Technologies Inc., CA, United States). High purity helium was offered as carried gas, and the flow rate was confirmed as 1.0 ml ∙ min^–1^. The column temperature was initially 40°C and heated to 230°C at a rate of 3°C/min for 3 min. The split ratio was 10:1 and the injection temperature was set to 260°C.

#### Nuclear Magnetic Resonance Analysis

*Chuanminshen violaceumis* polysaccharide sample (20 mg) was dissolved in 0.5 ml D_2_O for nuclear magnetic resonance (NMR) analysis. ^1^H, ^13^C, ^1^H-^1^H, Correlation Spectroscopy (COSY), Heteronuclear Singular Quantum Correlation (HSQC), and Heteronuclear Multiple Bond Correlation (HMBC) spectra were recorded on a Bruker Ascend 600 MHz Spectrometer.

### Animal and Experimental Design

C57BL/6 mice (male, 18–22 g, 6–8 weeks) were obtained from Changzhou Cavens Laboratory Animal Co., Ltd. (Changzhou, Jiangsu Province, China). The operating procedures were in strict accordance with the NIH guidelines for the care and use of laboratory animals (NIH Publication No. 8023, revised 1978). All experiments that were conducted followed the regulations for the Care and Use of Laboratory Animals of the National Institute of Animal Health and the Guidance by the ethics committee of Qingdao University (animal welfare assurance number: 14-0027).

For the I/R model, mice were randomly divided into four groups (*n* = 8 per group): Sham group, I/R group, CVP [10 mg kg^–1^, intragastric (i.g.)] groups, and CVP (20 mg kg^–1^, i.g.). The mice were pretreated with CVP for 1 h intragastrically, then the left anterior branch of descending coronary artery was occluded by a knot to create ischemia for 45 min followed by 24 h of reperfusion. Finally, blood samples were collected from the abdominal aortic artery, hearts and kidneys were isolated for the following experiments.

### 2, 3, 5-Triphenyltetrazolium Chloride and Evans Blue Double-Staining

After 24 h of reperfusion, 2.0% Evans blue dye was injected into the jugular vein of the heart. Then hearts were rapidly excised and sectioned into 1-mm-thick sections. Then slices were incubated in 1.0% 2, 3, 5-triphenyltetrazolium chloride (TTC) (Sigma-Aldrich, United States) for 15 min at 37°C. The areas of infarction (INF) and non-ischemic left ventricle (LV) were assessed by computerized planimetry (NIH Image 1.57).

### H&E Staining

Heart and kidney tissues were harvested and fixed in 10% formaldehyde. Then tissues were embedded in paraffin and sliced. After dehydration in gradient concentrations of alcohol, the sections were stained with H&E according to the instruction and then imaged using a light microscope.

### Detection of Blood Parameters

Blood samples were collected before the sacrifice of the mice and the levels of serum superoxide dismutase (SOD), glutathione peroxidase (GSH-PX), malondialdehyde (MDA), lactate dehydrogenase (LDH), creatine kinase-MB (CK-MB), cTnI, TNF-α, IL-6, creatinine, and blood urea nitrogen (BUN) were measured with related biochemical kits.

### Cell Culture and Anoxia/Reoxygenation Injury Model

H9c2 cells (American Type Culture Collection) were cultured in DMEM supplemented with 10% FBS, 100 U/ml penicillin, 100 μg/L streptomycin, and 110 mg/L sodium pyruvate in a humidified atmosphere containing 5% CO_2_ at 37°C. To mimic myocardial I/R injury, H9c2 cells (70% confluence) were plated into glucose- and serum-free DMEM in a hypoxia airtight gas chamber containing 95% N_2_ and 5% CO_2_ gas mixture at 37°C for 12 h. Subsequently, the medium was replaced with normal DMEM and cells were cultured for 6 h at 37°C with 5% CO_2_.

### Western Blotting Analysis

After cell lysis, equivalent protein amounts were separated by sodium dodecyl sulfate–polyacrylamide gel electrophoresis (SDS-PAGE) and transferred to a polyvinylidene difluoride (PVDF) membrane. After fixation and blocking, membranes were incubated with primary antibodies overnight at 4°C. After washing, membranes were incubated with secondary antibodies for 2 h at room temperature. Finally, chemiluminescence signals were detected with an enhanced ChemiDocTM Imager.

### Determination of Intracellular Iron and Lipid Peroxidation Levels

After treatment, H9c2 cells from 24-well plates were incubated with 1 μM FerroOrange in dark at 37°C for 30 min. Then the fluorescence was visualized under an inverted fluorescence microscope. In addition, cells from 6-well plates were stained with 10 μM C11 BODIPY 581/591 for 30 min in the dark at 37°C and then harvested by centrifugation at 1,500 rpm for 5 min. The lipid peroxidation level was analyzed by flow cytometry.

### Statistical Analysis

All assays were carried out at least in triplicate and the results were reported as means ± standard deviation (SD). Statistical comparisons were performed by using one-way ANOVA analysis with SPSS 19.0 software. Data significance was set at *p* < 0.05.

## Results

### Polysaccharides’ Preparation and Physicochemical Analysis

**FIGURE 1:**
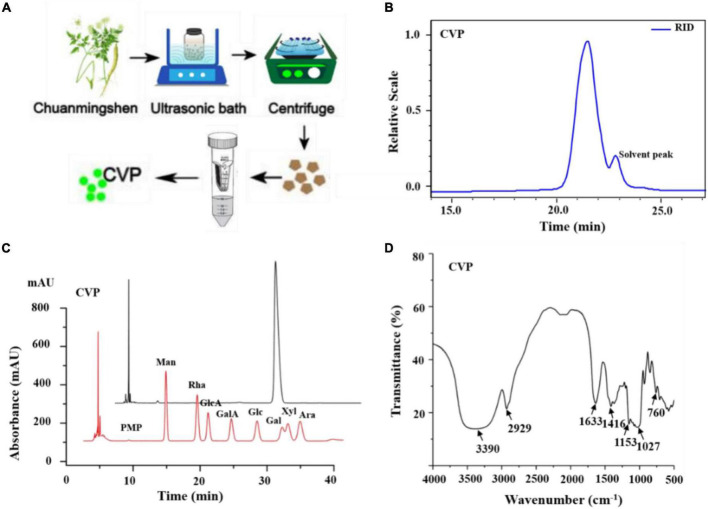
The preparation of *Chuanminshen violaceumis* polysaccharide (CVP) **(A)**; determination of CVP’s molecular weight **(B)**; the monosaccharide compositions of CVP **(C)**; and IR spectra **(D)**.

Crude polysaccharides were isolated from *Chuanminshen violaceum*’s root, and redundant proteins were removed (37, 38). Finally, we acquired one polysaccharide, namely, CVP through a series of purifying technologies (Figure 1A).

### Structure Characterization of *Chuanminshen violaceumis* Polysaccharide

#### *Chuanminshen violaceumis* Polysaccharide’s Molecular Weight and Compositional Monosaccharides

*Chuanminshen violaceumis* polysaccharide’s molecular weight was calculated to be 1.703 × 10^3^ Da with Gel Permeation Chromatography (GPC) and the curve reflected the acquisition of homogeneous polysaccharides (Figure 1B). Then CVP was hydrolyzed into a series of monosaccharides and derivatives were detected by HPLC. As shown in Figure 1C, the retention time of each peak reveals CVP’s monosaccharide composition. After analysis and calculation, CVP was found to be mainly composed of glucose.

#### Methylation Analysis of *Chuanminshen violaceumis* Polysaccharide

After obtaining CVP’s composition, we further characterized its glycosidic linkage between monosaccharides with methylation technology. Methylated CVP was hydrolyzed, acetylated, and analyzed by GC-MS as described previously (39). As shown in Table 1, monosaccharides are linked to each other by different glycosidic bonds, such as 1,4,5-tri-O-acetyl-2,3,6-tri-O-methyl glucitol standing for 4-Glcp. Based on this analysis, different patterns of the glycan chain information were obtained.

**TABLE 1:** Gas chromatography-mass spectrum (GC-MS) analysis of methylated alditol of *Chuanminshen violaceumis* polysaccharide (CVP) polysaccharide (detailed information had been shown in Supplementary Figure 2).

Linkage	Methylated glycosides	Molecular weight	Retention time (min)	Molar ratio (%)
T-Glc*p*	1,5-di-*O*-acetyl-2,3,4,6-tetra-*O*-methyl glucitol	323	9.740	25.85
3-Glc*p*	1,3,5-tri-*O*-acetyl-2,4,6-tri-*O*-methyl glucitol	351	13.038	6.54
4-Glc*p*	1,4,5-tri-*O*-acetyl-2,3,6-tri-*O*-methyl glucitol	351	15.105	42.77
3,4-Glc*p*	1,3,4,5-tetra-*O*-acetyl-2,6-di-*O*-methyl glucitol	379	17.220	10.78
4,6-Glc*p*	1,4,5,6-tetra-*O*-acetyl-2,3-di-*O*-methyl glucitol	379	19.320	10.71
3,4,6-Glc*p*	1,3,4,5,6-penta-*O*-acetyl-2-*O*-methyl glucitol	407	21.715	3.35

#### Fourier Transform Infrared and Nuclear Magnetic Resonance Analyses of *Chuanminshen violaceumis* Polysaccharide

Infrared spectrum of CVP was observed. The results exhibited strong bands at 3,390 cm^–1^ corresponding to O-H stretching vibrations, a band at 2,929 cm^–1^ corresponding to C-H stretching vibrations, and a strong band at 1,416 cm^–1^ to C-H bending vibrations (Figure 1D).

**FIGURE 2:**
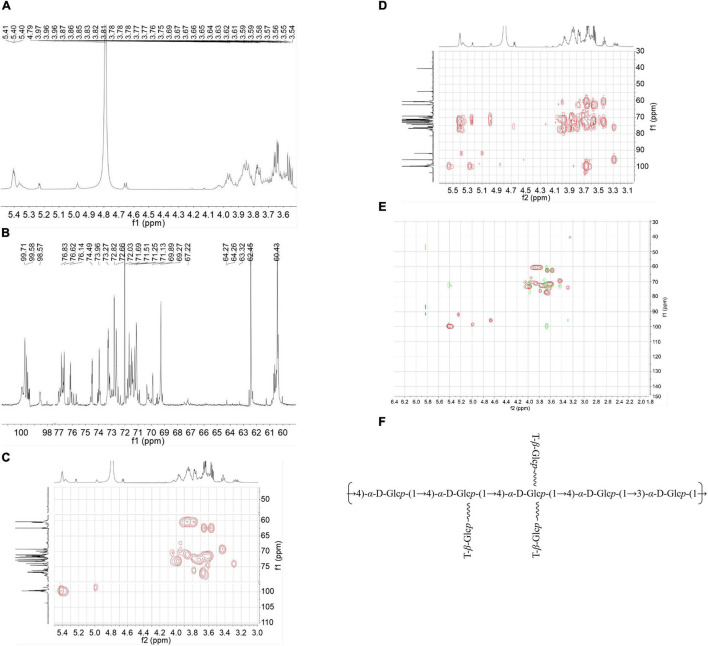
The nuclear magnetic resonance (NMR) spectra: H-NMR **(A)**, C-NMR **(B)**, Heteronuclear Singular Quantum Correlation (HSQC) **(C)**, Heteronuclear Multiple Bond Correlation (HMBC) spectra **(D)**, an overlap of *Chuanminshen violaceumis* polysaccharide (CVP) recorded in D_2_O **(E)**, and supposed structure of CVP **(F)**.

Subsequently, a set of NMR spectroscopy measurements were carried out to further characterize the structure of CVP. Data from ^1^H NMR spectroscopy showed that the main anomeric proton signals are from 4.9 to 5.5 ppm that included δ 4.98, 5.37, 5.39, 5.40, 5.41, and 5.42 ppm (Figure 2A).

The ^13^C NMR spectrum showed that the corresponding anomeric carbons are at δ 98.55, 99.35, 99.49, 99.71, 99.58, and 99.4 ppm (Figure 2B). According to the results of methylation and NMR spectra, proton and carbon signals were confirmed at δ 61.17/3.74, 3.66; δ 61.09/3.74, 3.64; and δ 60.41–60.45/3.76–3.80.

Then, we analyzed HMBC, HSQC, and COSY spectra for the other proton and carbon signals. In the HSQC spectrum, it presented the C-H signal at δ 99.71 and 5.40 (Figure 2C). As for the COSY (Supplementary Figure 1) and HMBC spectra (Figure 2D), we concluded the signals from H-1 at δ 5.40 ppm to H-2 at δ 3.64 ppm and H-3 at δ 3.97 ppm, from H-3 to C-4 at δ 73.9 ppm to H-4 at δ 3.66 ppm and C-6 at δ 60.6 ppm. According to other information, we speculated that these signals were attributed to →4)-α-D-Glcp-(1→ linkage. Other residues were subsequently confirmed by using a similar way.

After that, we analyzed the HMBC spectrum. From this result, repeated units of →4)-α-D-Glcp-(1→4)-α-D-Glcp-(1→ were verified by the correlation signals at δ H/δ C 5.40/76.6 ppm. Meanwhile, the existence of the linkage of →4)-α-D-Glcp-(1→3)-α-D-Glcp-(1→ was indicated by the correlation signals at δ H/δ C 5.40/76.2 ppm. Then we get the overlap of HSQC and HMBC as shown in Figure 2E. In addition, we inferred the chemical shifts of the residues as shown in Table 2. Finally, based on the above chemical information and many references, we propose a possible structure of CVP in Figure 2F.

**TABLE 2:** H-NMR and C-NMR spectra’s chemical shift of *Chuanminshen violaceumis* polysaccharide (CVP).

Residues	chemical shift (δ in ppm and *J* in Hz)						
		1	2	3	4	5	6
A →3)-α-Glc*p*-(1→	H	5.42	3.60	3.78	3.62	3.85	3.86, 3.78
	C	99.4	71.6	76.2	70.3	71.1	60.52
B →4)-α-Glc*p*-(1→	H	5.40	3.64	3.97	3.66	3.85	3.84, 3.78
	C	99.71	72.0	73.9	76.6	71.5	60.6
C →4,6)-α-Glc*p*-(1→	H	5.37	3.64	3.98	3.69	3.87	3.55, 3.65
	C	99.35	71.7	74.0	76.8	71.8	64.3
D →3,4)-α-Glc*p*-(1→	H	5.39	3.59	3.78	3.62	3.85	3.75, 3.83
	C	99.49	71.4	76.7	76.6	71.2	60.8
E →3,4,6)-α-Glc*p*-(1→	H	4.98	3.62	3.78	3.61	3.88	3.66, 3.54
	C	98.55	71.4	76.7	76.6	71.4	63.3
F T-β-Glc*p*-(1→	H	4.66	3.27	3.63	3.44	3.54	3.86, 3.78
	C	103.6	76.3	77.0	72.6	75.9	62.6

### *Chuanminshen violaceumis* Polysaccharide Protected Myocardial Ischemia-Reperfusion Injury in Mice

**FIGURE 3:**
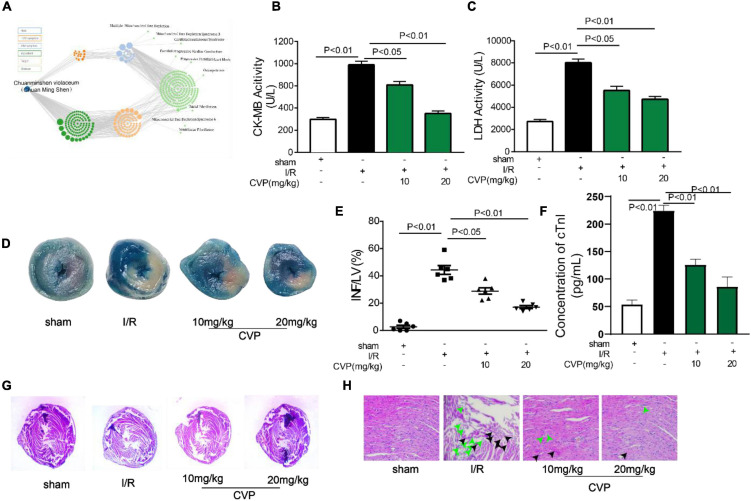
The protective effect of CVP on I/R injury in mice. Results of network pharmacology analysis of *Chuanminshen violaceum* **(A)**; mice were exposed to 45 min ischemia and 24 h reperfusion to construct the I/R model. Then serum levels of CK-MB **(B)**, LDH **(C)**, and cTnI **(F)** were measured with biochemical test kits; after hearts were separated, infarct size was estimated by TTC and Evans blue doubling-staining, blue represents normal tissue, red represents ischemic but non-infarcted areas, and white represents ischemic and infarcted areas **(D)**; infarct size was quantized using computerized planimetry **(E)**; morphological changes of myocardium **(G)** and myocardial cells **(H)** were observed by H&E staining, the green arrow represents damaged myocardial cells and the black arrow represents myocardial edema. cTnI, cardiac troponin I; CVP, *Chuanminshen violaceumis* polysaccharides; INF, infarction; I/R, ischemia-reperfusion; LDH, lactate dehydrogenase; LV, left ventricle.

Based on our network pharmacology prediction that *Chuanminshen violaceum* has the potential for preventing heart diseases (Figure 3A), CVP’s effect on myocardial I/R was explored. Firstly, myocardial reperfusion injury had occurred in I/R mice evidenced by MI area formation (Figures 3D,E), cardiac muscle necrosis markers CK-MB (Figure 3B), LDH (Figure 3C), and cTnI (Figure 3F) overleakage, as well as morphological changes in cardiac myocytes (membranolysis and cellular edema) (Figures 3G,H) in I/R mice. Noticeably, the above phenomena were significantly alleviated with CVP pretreatment in a dose-dependent manner.

### *Chuanminshen violaceumis* Polysaccharide Inhibited Oxidative Stress and Inflammation

**FIGURE 4:**
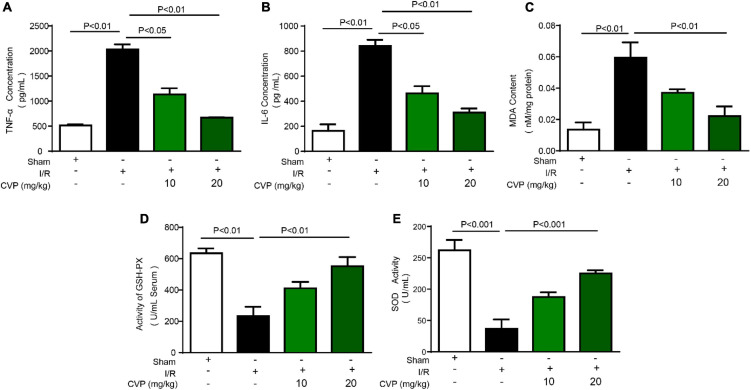
*Chuanminshen violaceumis* polysaccharide (CVP) inhibited oxidative stress and inflammation response in I/R mice. After serum was collected from the abdominal aortic artery, levels of pro-inflammatory cytokines TNF-α **(A)** and IL-6 **(B)** were detected with ELISA kits, oxidative stress markers MDA **(C)**, GSH-PX **(D)**, and SOD **(E)** were evaluated by biochemical detection of serum. CVP, *Chuanminshen violaceumis* polysaccharides; GSH-PX, glutathione peroxidase; IL-6, interleukin-6; I/R, ischemia-reperfusion; MDA, malondialdehyde; SOD, superoxide dismutase; TNF-α, tumor necrosis factor-α.

In I/R mice, decreased GSH-PX and SOD activity (Figures 4D,E), as well as increased MDA production (Figure 4C), were observed while CVP significantly rebalanced these changes indicating CVP’s anti-oxidative stress activity. Noticeably, high circulating levels of pro-inflammatory markers, TNF-α (Figure 4A) and IL-6 (Figure 4B), were evaluated in large releases in I/R mouse serum and decreased with CVP pretreatment demonstrating its beneficial anti-inflammatory activity.

### *Chuanminshen violaceumis* Polysaccharide Protected Acute Renal Injury

**FIGURE 5:**
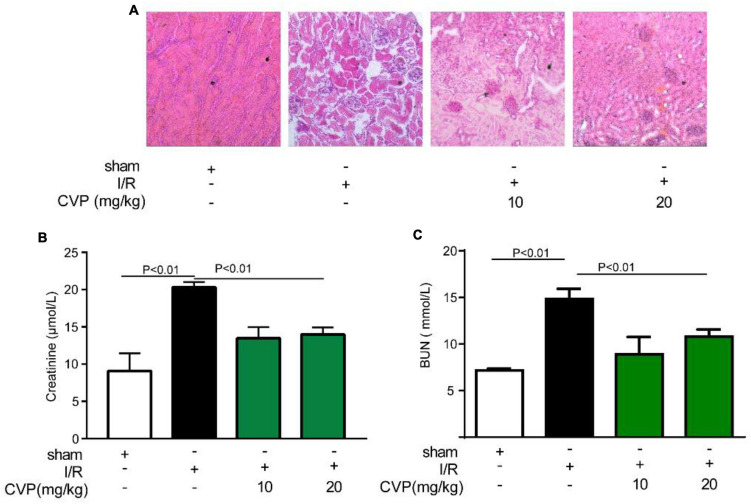
The effect of CVP on acute renal injury secondary to I/R injury. After the kidney was isolated, morphological alterations of the kidneys were examined *via* H&E staining (×10) **(A)**; the renal function was assessed by detecting the levels of blood creatinine **(B)** and BUN **(C)**. BUN, blood urea nitrogen; CVP, *Chuanminshen violaceumis* polysaccharides; I/R, ischemia-reperfusion.

In the study, CVP’s protective effect on kidney injury was further studied. Data showed that CVP significantly decreased serum renal function parameters, BUN, and creatinine levels, which were accumulated in I/R mice (Figures 5B,C). Additionally, based on H&E staining assay results, CVP obviously attenuated tubular injury by alleviating glomerular atrophy and reducing vacuolization (Figure 5A). The results proved that CVP has a potential protective effect on nephrotoxicity secondary to myocardial I/R injury.

### Ferroptosis Was Occurred in Anoxia/Reoxygenation-Induced Cardiomyocyte Death and Restrained by *Chuanminshen violaceumis* Polysaccharide Pretreatment

**FIGURE 6:**
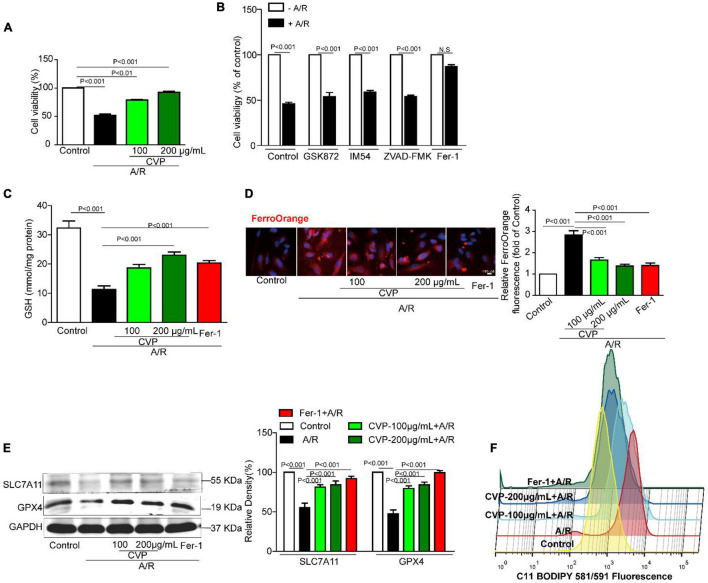
The effect of CVP on A/R-stimulated cardiomyocytes and related mechanisms *in vitro*. Cardiomyocytes were pretreated with necroptosis inhibitor GSK872 (10 μM), apoptosis inhibitor ZVAD-FMK (1 μM), necrotic inhibitor IM54 (10 μM), ferroptosis inhibitor Fer-1 (10 μM), and CVP (200 μg/ml) for 1 h and then stimulated with A/R, cell viability was tested with MTT assay **(A,B)**; cellular GSH concentration was detected by GSH Detection Assay Kit **(C)**; intracellular Fe^2+^ was assessed with dye FerroOrange **(D)**; SLC7A11 and GPX4 were detected by Western blotting **(E)**; and fluorometric analysis of lipid ROS was carried out using C11-BODIPY dye **(F)**. A/R, anoxia/reoxygenation injury; CVP, *Chuanminshen violaceumis* polysaccharides; Fer-1, ferrostatin-1.

In *in vitro* experiment, H9c2 cells were damaged with anoxia/reoxygenation (A/R) to mimic I/R injury and ameliorated with CVP pretreatment (Figure 6A). Furthermore, exact death manners and mechanisms were explored. Identified by a series of death inhibitors, cell viability was not significantly improved by necrosis inhibitor IM54, necroptosis inhibitor GSK872, and apoptosis inhibitor ZVAD-FMK, in contrast, ferroptosis inhibitor Fer-1 apparently restored cell viability by implying the occurrence of ferroptosis in A/R-induced H9c2 death (Figure 6B). Subsequently, a series of ferroptosis indexes that include iron overload, lipid peroxidation, and antioxidant axis SLC7A11-GSH-GPX4, were detected. Data from Figures 6C–F show that in damaged H9c2 cells, cellular Fe^2+^ overload and lipid ROS are gradually increased with a decrease in GPX4 and SLC7A11 protein expression levels and GSH efflux. Meanwhile, CVP reversed the above phenomena to suppress ferroptosis.

## Discussion

Nowadays, more and more novel polysaccharides were extracted from natural plants and were confirmed to possess multiple biological activities (40). In our study, one polysaccharide, namely, CVP was isolated from *Chuanminshen violaceum*’s root, one well-known tonic food and traditional Chinese medicine. Subsequently, CVP’s molecular weight (1.703 × 10^3^ Da), compositional monosaccharides (Figure 1C), and structure (Figure 2F) were confirmed. Considering that plenty of polysaccharides were reported to own potential for treating cardiovascular diseases, in the following study, CVP’s protective effects on myocardial damage caused by I/R were explored. As shown in Figure 3, CVP significantly reduces MI area, decreases serum levels of cardiac muscle necrosis markers, CK-MB, LDH, and cTnI, and restores morphological changes of damaged cardiomyocytes occurred in I/R mice. The above phenomena indicated the protective effect of CVP on I/R-induced myocardial damage.

Oxidative stress and inflammation are pivotal causes in response to severe reperfusion injury. Therefore, possessing antioxidant and anti-inflammatory activities is the dominant properties of the ideal drug to treat reperfusion injury. As shown in Figure 4, CVP effectively reduces the serum level of MDA and improves the activities of GSH-PX and SOD in I/R mice. In addition, CVP significantly reduced the secretions of inflammatory factors, TNF-α and IL-6. Moreover, kidney injury is one serious complication accompanied by acute MI (41) and also a serious adverse reaction of clinical therapeutic agents. In our study, minimal renal toxicity of CVP-treated mice was also demonstrated. The above data indicated that CVP is one promising therapeutic candidate for I/R injury.

Up to now, exact death manners and related mechanisms during the process of myocardial reperfusion injury are confused. Recent studies have revealed that ferroptosis plays a crucial role in the pathogenesis of various cardiovascular diseases (42, 43). In the current study, A/R injury provoked enhanced ferroptosis in H9c2 myocytes while both CVP and ferroptosis inhibitors Fer-1 restore the survival rate of cells. Then indicators related to ferroptosis that include Fe^2+^, GSH, GPX4, and SCL7A11 were also detected. As shown in Figure 6, Fe^2+^ overload and GSH consumption are observed in the model group while CVP restores the balance. Then SLC7A11/GPX4 pathway was activated by CVP to confront A/R-induced ferroptosis. Moreover, iron overload-promoted lipid ROS was generated largely with A/R injury and obviously reduced with CVP treatment. The above data revealed CVP’s potential protective mechanisms. Different from targeted medicines, multi-target and multi-channel are the treatment characteristics for natural products. As the extraction from *Chuanminshen violaceumis*’ dry root, maybe CVP has the advantage of multi-target therapy. GSH/GPX4/SLC7A11 pathway was CVP’s potential multi-target regulatory pathway.

## Conclusion

In summary, our study identified one polysaccharide, namely, CVP and evidenced its cardioprotective effects. *In vivo*, CVP demonstrated prominent therapeutic advantages in preventing myocardial I/R. *In vitro*, inhibition of ferroptosis was proved the potential therapeutic mechanism for CVP alleviating myocardial damage in response to A/R damage. Overall, our study offers a potential drug candidate and novel therapeutic target for treating myocardial reperfusion injury.

## Data Availability Statement

The original contributions presented in this study are included in the article/Supplementary Material, further inquiries can be directed to the corresponding authors.

## Ethics Statement

The animal study was reviewed and approved by the Ethics Committee of Qingdao University.

## Author Contributions

PH, MiZ, and MeZ planned and performed experiments, analyzed the data, and drafted the manuscript. MYZ, HL, and YH helped to perform *in vivo* experiments. BM and DW helped to perform *in vitro* experiments. ZZ and WZ conceived the scientific ideas, oversaw the project, designed the experiments, and refined the manuscript. All authors contributed to the article and approved the submitted version.

## Conflict of Interest

The authors declare that the research was conducted in the absence of any commercial or financial relationships that could be construed as a potential conflict of interest.

## Publisher’s Note

All claims expressed in this article are solely those of the authors and do not necessarily represent those of their affiliated organizations, or those of the publisher, the editors and the reviewers. Any product that may be evaluated in this article, or claim that may be made by its manufacturer, is not guaranteed or endorsed by the publisher.
