# Supplementary figures and images for: A Novel Polysaccharide From Chuanminshen violaceum and Its Protective Effect Against Myocardial Injury
Source: Front Nutr. 2022 Jul 14;9:961182. doi: 10.3389/fnut.2022.961182 (PMC9330552; doi:10.3389/fnut.2022.961182)

---

## Supplementary data

Supplementary Fig.1

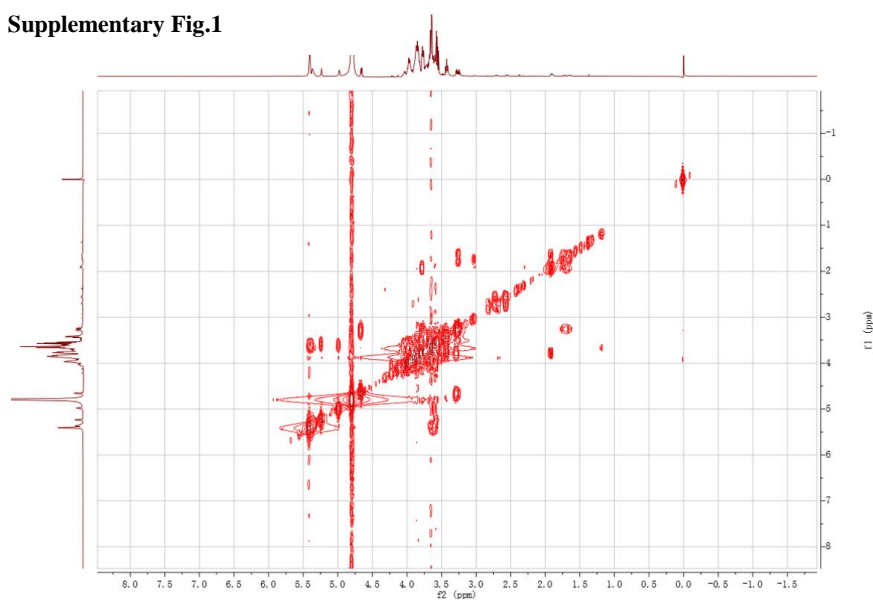

Supplementary Fig.1 COSY spectra of CVP

Supplement: Supplementary file 1 [file Data_Sheet_1.pdf]
