# Supplementary material for: A Novel Polysaccharide From Chuanminshen violaceum and Its Protective Effect Against Myocardial Injury
Source: Front Nutr. 2022 Jul 14;9:961182. doi: 10.3389/fnut.2022.961182 (PMC9330552; doi:10.3389/fnut.2022.961182)

Supplymentary Fig.2

Total ions chromatogram of CVP derivate

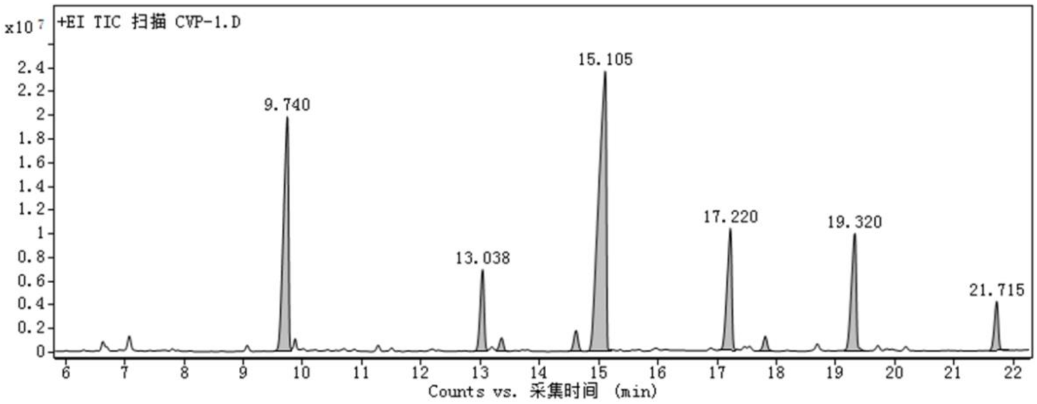

Fragment 1

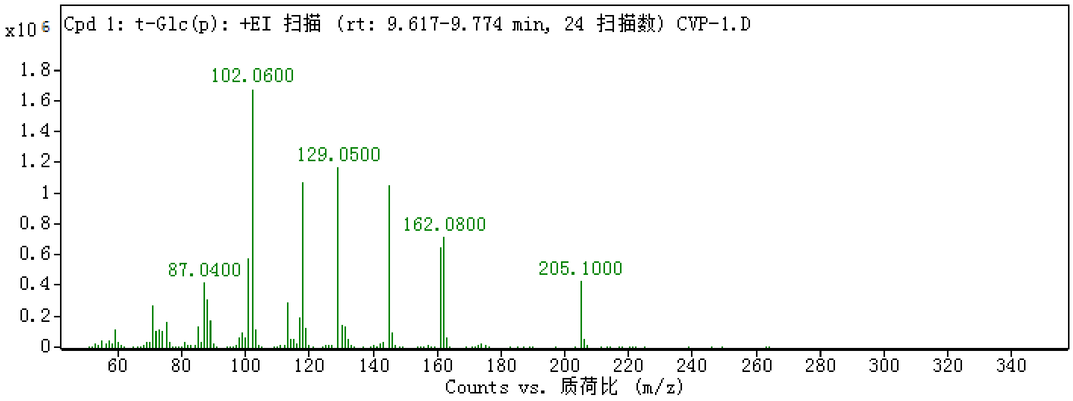

Fragment 2

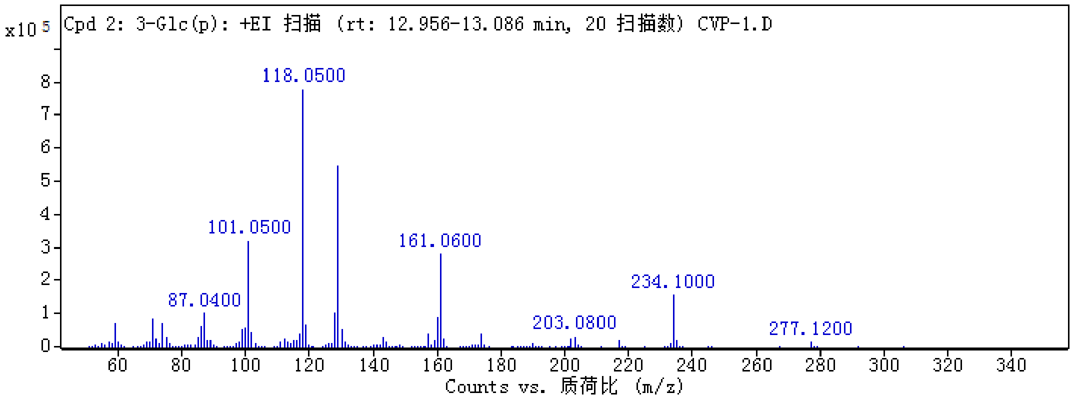

Fragment 3

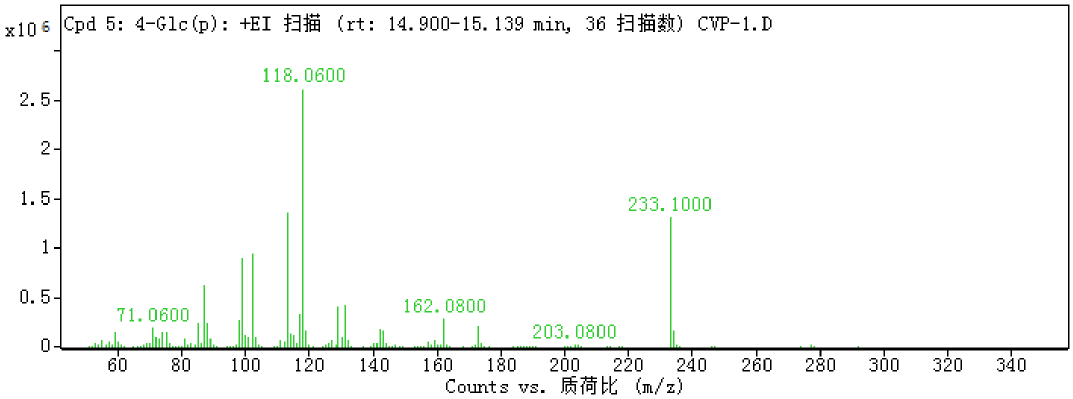

Fragment 4

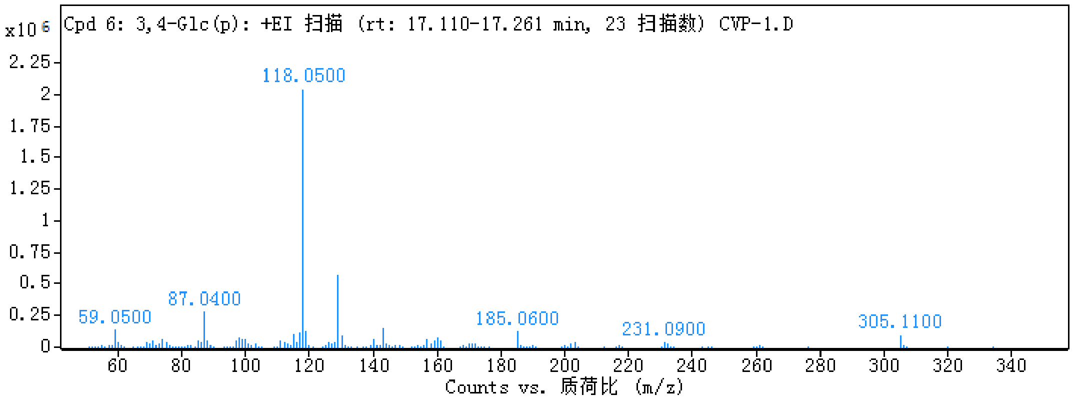

Fragment 5

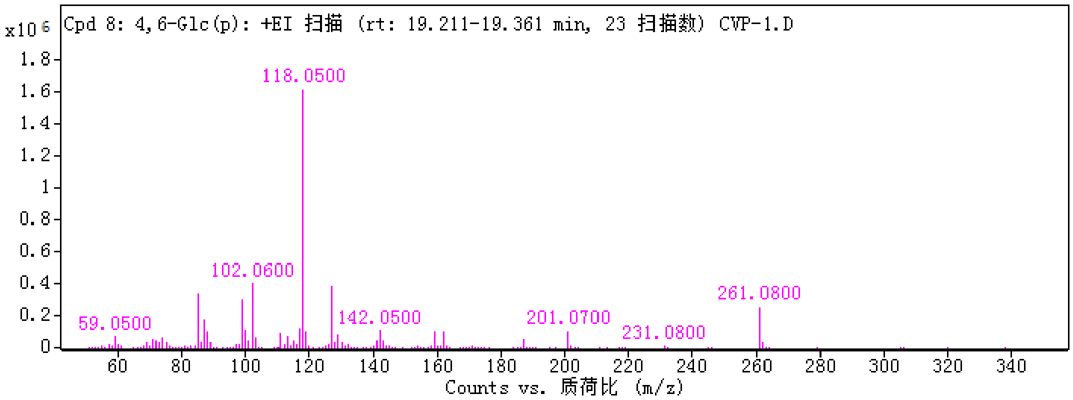

Fragment 6

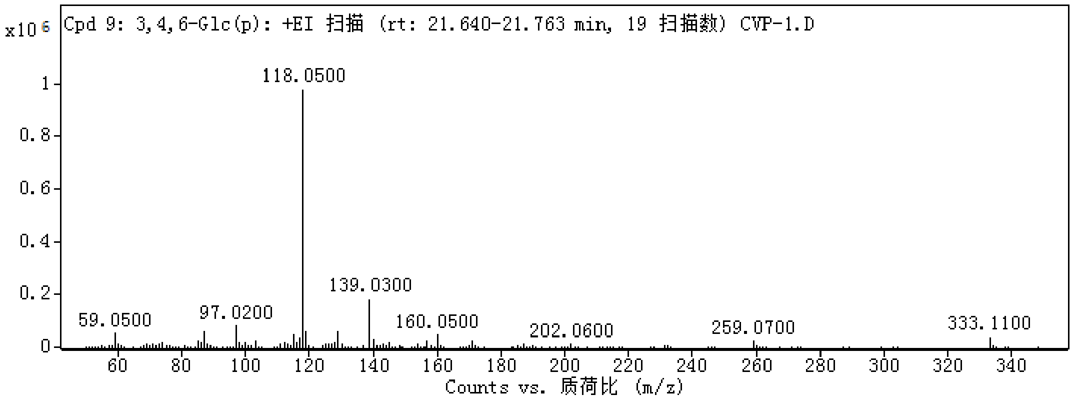

Supplement: Supplementary file 2 [file Data_Sheet_2.PDF]
